# Loneliness and Bullying by Siblings in Gender-Diverse Adolescents: Results From the Population-Based Generation R Study

**DOI:** 10.1016/j.jaacop.2026.04.003

**Published:** 2026-04-23

**Authors:** Yllza Xerxa, Akhgar Ghassabian, Manon H.J. Hillegers, Luis Martinez Agulleiro, Pauline W. Jansen, Samantha Busa, Francisco Xavier Castellanos, Tonya White

**Affiliations:** a Erasmus University Medical Center, Rotterdam, the Netherlands; b The Generation R Study Group, Erasmus University Medical Center, Rotterdam, the Netherlands; c New York University Grossman School of Medicine, New York, New York; d Nathan Kline Institute for Psychiatric Research, Orangeburg, New York; e Erasmus University Rotterdam, Rotterdam, the Netherlands; f Section on Social and Cognitive Developmental Neuroscience, National Institutes of Mental Health, Bethesda, Maryland

**Keywords:** family risk factors, gender diversity, loneliness, social support

## Abstract

**Objective:**

Gender-diverse individuals often face a burden of poor mental health. This study examined whether gender-diverse experiences were associated with higher levels of loneliness in adolescents, over and above depression and anxiety, and how family environmental factors, including maladaptive parenting, being bullied by a sibling at home (victimization), and bullying a sibling at home (perpetration), moderate the associations between gender-diverse and loneliness experiences among 4,424 adolescents in a population-based cohort.

**Method:**

This cross-sectional study was embedded in Generation R, a multiethnic population-based cohort from fetal life onward. Adolescents with information on self-reported or parent-reported gender diversity and loneliness at ages 13 to 15 years were included.

**Results:**

Adolescents who reported gender diversity had higher levels of loneliness compared with youth who did not report gender diversity (assigned female at birth: *B* = .67, 95% CI 0.49-1.23; assigned male at birth: *B* = .45, 95% CI 0.32-0.98). The associations remained after taking into account depression and anxiety, indicating a unique association of gender diversity with lonely feelings in adolescence (assigned female at birth: *B* = .38, 95% CI 0.24-0.51; assigned male at birth: *B* = .31, 95% CI 0.17-0.46). Being bullied by a sibling at home (victimization) modified the association of gender diversity with loneliness experiences (interaction *p* = .04). No evidence was found for interaction effects between gender diversity, maladaptive parenting, and any temperamental traits on adolescent loneliness (*p*s > .10).

**Conclusion:**

Gender diversity is associated with higher levels of loneliness in adolescents. Being a target of bullying modified the association of gender diversity with loneliness experiences, suggesting that gender-diverse adolescents who are bullied by siblings experience particularly higher levels of loneliness.

## Plain language summary

Gender-diverse youth experience substantial mental health disparities when compared to cisgender youth. This cross-sectional study examined how gender diversity related to loneliness in adolescents, finding higher loneliness levels among gender-diverse youth, independent of depression and anxiety. Sibling bullying—not maladaptive parenting or temperamental traits—was associated with higher levels of loneliness. These findings emphasize modifiable risk factors and the need for targeted support in family and social environments.

Gender diverse refers to individuals whose gender identity, the way they identify and/or express themselves, is different from their sex assigned at birth.^1^ Gender-diverse individuals commonly experience emotional and behavioral problems,^2^, ^3^, ^4^, ^5^ and thus it is crucial to understand the relation of gender diversity with developmental neurobiology^6^ and manifestation of emotional and behavioral disorders. According to the minority stress theory, chronic internal and external stressors (eg, internalized transphobia, perceived stigma, discrimination, or rejection) experienced by gender-diverse people contribute to mental and physical health problems,^7^ such as depression.^8^ This stress can translate into disparities in multiple domains of life, including poor health, education, and social outcomes.^9^ Gender-diverse individuals face a burden of poor mental health, with quantitative and qualitative evidence suggesting that loneliness and social isolation are key mechanisms.

Loneliness is an important factor in the mental health outcomes of gender-diverse individuals. Research suggests that loneliness mediates the relation between minority stress and depression in gender-diverse populations.^10^^,^^11^ For transgender youth, internalized transphobia, loneliness, and social support are key factors in psychological well-being.^12^ Additionally, studies have highlighted the role of loneliness in the mental health of gender minorities, emphasizing its impact with other stressors, such as discrimination and social isolation.^13^, ^14^, ^15^ These findings underscore the importance of not only evaluating loneliness in gender-diverse adolescents, but also implementing interventions to reduce loneliness in this marginalized population.

Conversely, social support factors, such as positive peer relationships, have been shown to promote resilience in gender-diverse individuals.^16^^,^^17^ However, research has also shown that many gender-diverse individuals experience pervasive stigma related to their gender diversity.^7^ Moreover, risk factors such as maladaptive parenting and sibling bullying, as well as temperamental traits, such as negative affectivity, surgency (ie, a tendency toward high positive affect, spontaneity, and sociability), and effortful control (ie, the ability to regulate attention and behavior voluntarily), may interact with gender diversity to influence and shape child development. The combination of gender diversity and loneliness during adolescence needs careful consideration in research and policy and should incorporate the cultural context for prevention and intervention.

Recently, there has been greater attention worldwide toward gender diversity, both negative and positive. Despite some of positive attention promoting greater acceptance, gender-diverse individuals remain a socially marginalized group. Because of persistent marginalization and isolation, these individuals can experience loneliness. Loneliness, defined as a distressing emotional state that arises from the discrepancy between one’s perceived and desired levels of social connection,^18^ is a major risk factor for psychological disturbances and poor health outcomes in adolescents.^19^^,^^20^ As such, even in the absence of social separation, the feeling of loneliness may result from unfriendly environments that lack compassionate care and help. Loneliness is, by definition, a subjective, internal state of mind. In adults, perceived loneliness is associated with a substantial increased risk of premature mortality, independent of income, education, sex, and ethnicity.^21^^,^^22^ Research has yielded broadly consistent findings on developmental patterns of loneliness in childhood and adolescence.^20^^,^^23^, ^24^, ^25^ These studies indicate that individuals who experience either long-term high or average levels of loneliness have poorer mental health outcomes compared with individuals who do not experience loneliness. Loneliness has been linked to psychological consequences, such as depression, anxiety, and impaired social functioning (eg, see Weiss^26^ and Peplau and Perlman^27^), highlighting the importance of understanding its developmental trajectory and correlates in adolescence. Furthermore, despite considerable research on loneliness in the general population, studies assessing the relation between gender-diverse experiences and loneliness in adolescence are limited.

Research on gender-diverse individuals (nationally representative samples) showed that loneliness was higher in transgender and gender-diverse people living in Norway,^28^ the United States,^29^ and Australia^30^ compared with cisgender peers. However, these studies did not include children and adolescents from the general population. Population-based samples can include not only help-seeking youth, but also those on the broader spectrum of gender diversity.

Emerging evidence suggests that family environments are powerful determinants of LGBTQ (lesbian, gay, bisexual, transgender, queer) youth mental health. Pioneering work by the Family Acceptance Project found that familial rejection increases suicide attempt risk more than 8-fold and amplifies depression, substance use, and health risk behaviors.^31^ By contrast, family acceptance predicts better self-esteem, social support, and reduced psychopathology. Consistent with findings from the Family Acceptance Project, family rejection during adolescence is associated with an 8-fold increase in suicide attempt risk and elevated depression and substance use.^32^ Specific supportive behaviors by parents, such as respectful dialogue and standing up for their child, are linked to significantly lower odds of suicide among LGBTQ youth.

In the gender diversity and child development literature, family risk and protective factors are another missing element that has rarely been examined.^33^ Child emotional and behavioral problems are often the result of maladaptive parenting or interparental conflict,^34^ a risk factor for the persistence of poor development and well-being.^35^ There are a growing number of studies within the field of child development describing risk and protective factors in gender-diverse youth.^36^, ^37^, ^38^, ^39^ These studies show the critical role of family support and acceptance in promoting mental health and well-being. Family support can serve as a buffer against enacted stigma and adverse outcomes and fosters resilience among transgender and gender-diverse adolescents. Research suggests that psychological and social benefits gained from being part of a community may be particularly important in gender-diverse individuals when experiencing minority stress.^40^^,^^41^

Temperamental traits can interact with gender diversity and influence adolescent loneliness and well-being. As such, temperament is believed to have direct effects—for instance, high negative affectivity may heighten loneliness by intensifying emotional distress—and indirect effects by shaping the social environment—for instance, high negative affectivity may elicit peer rejection or parental concern. Temperament also interacts with social and environmental experiences, exacerbating or buffering their effects.^42^ The interplay between gender diversity and child temperament in each of these mechanisms might be particularly relevant in understanding the association between child temperament and loneliness. We hypothesize that temperamental traits moderate the relation between gender diversity and loneliness, such that gender-diverse adolescents with more reactive or less adaptive temperaments experience higher levels of loneliness compared with cisgender peers.

Adolescence can be challenging for many individuals, and gender-diverse teens face unique social struggles. Although peers and outside activities become more prominent, the family remains an important support system. When the home, ideally a safe space, lacks acceptance, it can contribute to loneliness, which may worsen mental health outcomes.^43^ Understanding this is crucial for developing targeted interventions. The minority stress framework highlights how stigma, discrimination, and internalized transphobia increase loneliness, which can lead to mental health issues in gender-diverse youth. Research shows that these stressors create vulnerabilities,^3^^,^^44^ underscoring the need for specialized support.

Parental and peer acceptance, along with school environments, are crucial factors in understanding loneliness, but family dynamics also play a significant role.^45^^,^^46^ Maladaptive parenting and bullying can create hostile environments, intensifying feelings of loneliness and rejection experienced by gender-diverse youth already facing societal stigma.^39^ Although research on familial influences is limited, these factors highlight the importance of understanding how negative family interactions contribute to loneliness alongside external stigma.

Within a general pediatric population setting, we examined associations of gender-diverse experiences with loneliness in a large sample of 13- to 15-year-old adolescents. We had 3 aims. First, we examined whether gender-diverse experiences were associated with higher levels of loneliness in adolescents assigned male or female at birth, over and above depression and anxiety. Second, we investigated interaction effects of family environmental factors, including maladaptive parenting, being bullied by a sibling at home (victimization), and bullying a sibling at home (perpetration), with gender diversity in association with adolescents’ experiences of loneliness. Third, we investigated whether temperamental traits, such as negative affect, surgency, and effortful control, interacted with gender diversity in relation to loneliness. Gender-diverse youth include not only transgender and nonbinary individuals, but also those who are exploring or questioning their gender identity, including individuals who may wish to be treated as another gender but do not yet identify persistently with a gender different from the one assigned at birth. This distinction is important as the experiences of youth exploring gender identity can differ from the experiences of youth with a consistent, enduring gender identity.

## Method

### Participants

This research was embedded in Generation R, a multiethnic population-based cohort that follows children and their families from fetal life onward.^47^ Briefly, recruitment occurred prenatally between 2002 and 2006 in Rotterdam, the Netherlands. Children and their families were assessed repeatedly using surveys and in-person measurements. The study was approved by the Medical Ethics Committee of the Erasmus Medical Center, Rotterdam. Parents provided written informed consent for each phase of the study, and children provided assent beginning at 12 years of age.

**Figure 1:**
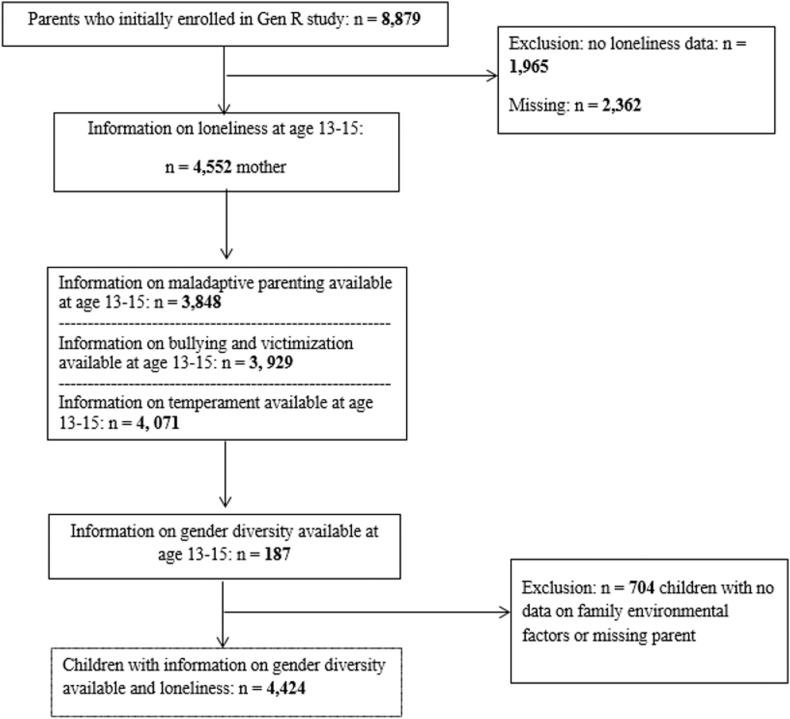
Inclusion Criteria of the Study Sample

The Generation R cohort originally enrolled 8,879 participants at baseline. At ages 13 to 15 years, 4,552 adolescents and/or their parents reported on gender diversity. Of these, 4,424 had complete data on gender diversity and loneliness and were included in descriptive analyses (Figure 1). Of the 4,424 adolescents, 112 (59.9%) were classified as low gender diversity and 75 (40.1%) were classified as high gender diversity. The analytic sample for regression models was restricted to adolescents with complete data on exposure and outcome variables. Within this group, 187 adolescents were identified as gender diverse, of whom 156 provided data on self-reported pubertal status (16.6% missing).

### Gender Diversity

We selected items from the Child Behavior Checklist for Ages 6 to 18 (CBCL/6-18, reported by parent) and the Youth Self-Report for Ages 11 to 18 (YSR/11-18, reported by child) of Achenbach System of Empirically Based Assessment (ASEBA)^48^^,^^49^ forms and the Gender Identity/Gender Dysphoria Questionnaire for Adolescents and Adults (GIDYQ-AA)^50^ to assess gender diversity in adolescents. The age range selected for this study (ages 13-15) was chosen based on the developmental context in which gender identity exploration is often socially and physically emphasized, particularly during high school years when adolescents are experiencing puberty and increasingly engaged in gendered socialization (Supplement 1, available online).

### Adolescent Loneliness

Loneliness was assessed by a collection of measures using the self-reported Brief Problem Monitor (BPM) at age 10 and the YSR/11-18^51^^,^^52^ at age 14. Youth reported on 4 items that measure loneliness: “I like being with people” (reverse coded); “I feel lonely”; “I would rather be alone than with others”; “Other boys or girls don’t like me.” A continuous loneliness scale was used in the analyses, which is the mean score of all items (Supplement 1, available online).

### Depression and Anxiety

Information on offspring depression and anxiety was obtained using the Brief Problem Monitor (BPM/11-18),^53^^,^^54^ a standardized child self-report of problem behaviors (ages 10 and 15). The BPM/11-18 is a validated abbreviated version of the Youth Self-Report (YSR/11-18).^51^^,^^52^ The depression and anxiety subscale comprised items such as “I am unhappy, sad, or depressed” and “I am too anxious or fearful.” For each subscale, we computed a continuous total score, which is the mean score of all items within the subscale (Supplement 1, available online).

### Maladaptive Parenting (Parent Report)

Maladaptive parenting was assessed by a collection of measures using the Overreactivity subscale from the Parenting Scale and using an adapted version of the Parent–Child Conflict Tactics Scale (CTSPC).^55^ The Overreactivity subscale was administered to measure emotional reactivity in the context of discipline encounters (eg, “Things build up and I do things I don’t mean to”; “I usually get into a long argument with my child”; “I give my child a long lecture”). Discipline tactics varied from explaining why something was wrong, distracting the child, punishing (eg, taking away privileges), and verbal or physical aggression. Example items are “I punished him/her by forbidding something that he/she wanted to do or have” and “I pinched his/her arm angrily” (Supplement 1, available online).

### Bullying and Victimization by Siblings

When children were 13 to 15 years old, they were asked to report their involvement in sibling bullying. Items were based on a sibling bullying questionnaire adapted from the Olweus Bullying Questionnaire,^56^ addressing bullying between brothers and sisters.^57^ Examples of sibling bullying were provided to children: a brother or sister says nasty and hurtful things to you; completely excludes you from their group of friends; hits, kicks, pushes, or shoves you; tells lies or spreads false rumors about you. They were then asked to report on their experience of sibling bullying within the last 6 months.

### Temperament

Child temperament was assessed by the Children’s Behavior Questionnaire–Very Short Form (CBQ-VSF)^58^ when children were 6 years old. The CBQ-VSF provides a highly differentiated assessment of temperament that has been validated across various studies.^58^ The CBQ-VSF consists of 36 items informing about child behavior in daily situations. The scores of negative affect, surgency, and effortful control were computed by averaging the item scores (Supplement 1, available online).

### Covariates

Date of birth (used to calculate a child’s age) and assigned sex at birth were obtained from birth records. Maternal and paternal ages were collected at enrollment during pregnancy. Maternal country of origin was categorized as Dutch or non-Dutch. Information on country of origin was obtained from female-identifying parents only, as these data were collected at enrollment during pregnancy and thus primarily reflect maternal background. Maternal education was classified in 3 levels: low (maximum of 3 years of general secondary school), medium (>3 years of general secondary school, intermediate vocational training), and high (bachelor’s degree or higher academic education). In questionnaires collected at the 13- to 15-year-old wave, puberty was assessed via self-report using the Puberty Developmental Scale,^59^ a validated self-report questionnaire with 5 items answered on a 5-point scale.

### Statistical Analysis

Descriptive statistics are presented for adolescent gender diversity at ages 13 to 15 years (mean = 14.1 years). In primary analyses, separate linear regressions were performed to test associations between adolescents’ report of gender diversity (stratified by child sex at birth)^60^^,^^61^ and loneliness experiences. Gender diversity was defined as the independent variable, and adolescent loneliness was defined as the dependent variable.

Then, we tested the interaction effects of family environmental factors, such as maladaptive parenting and bullying and victimization by siblings, with gender diversity on adolescent loneliness experiences. We also examined the interaction effects of each temperamental dimension (negative affect, surgency, and effortful control) with gender diversity on adolescent loneliness experiences.

Unstandardized regression coefficients (*B*) and 95% CIs were calculated based on the analytic subsample, which included participants with complete data on both gender diversity and loneliness. Descriptive statistics reported here are derived from the full Generation R cohort (N = 4,424). Models were adjusted for potential confounding variables, including maternal age, education, and country of origin and child’s age and Puberty Developmental Scale. Data on maternal education were missing for 215 participants (5.7%), country of origin data were missing for 73 participants (3.8%), and self-reported puberty data were missing for 735 participants (21.4%). In the gender-diverse subsample (n = 187), missingness on self-reported puberty was 16.6% (31/187). We performed multiple imputation that yielded unbiased parameter estimates using chained equations with 20 imputed datasets to account for missing values in potential confounders.^62^ Statistical analyses were performed using R version 4.1.2 (https://www.R-project.org/). This study followed the Strengthening the Reporting of Observational Studies in Epidemiology (STROBE) reporting guideline.

## Results

Descriptive statistics for participants’ characteristics are presented in Table 1. The mean (SD) age of the adolescents was 13.8 (0.64) years, and 48.6% of participants were assigned female at birth (AFAB). Of mothers, 38% were born in the Netherlands. As reported by both parents and children, 4.2% (187 of 4,424) of adolescents reported that they either sometimes or very often wished to be the opposite sex.^2^ Adolescents who were AFAB were more likely to report gender diversity compared with those assigned male at birth (AMAB), with 5.9% of AFAB adolescents vs 2.7% of AMAB adolescents reporting gender diversity (odds ratio = 2.27). The mean (SD) age of self-reported puberty score during assessment was 13.5 (0.8) years; mean (SD) age in AMAB adolescents was 13.7 (0.64) and in AFAB adolescents was 13.4 (0.61) years.

**Table 1:** Participant Characteristics (N = 4,424)

	Gender diversity at ages 13-15 years	
	**Mean**	**(SD)**
Child age at assessment of gender diversity, y	13.8	(0.6)
	**n**	**(%)**
Assigned sex at birth, female	1,547	(48.6)
	**Mean**	**(SD)**
Child self-report puberty scale score at age 13-15 y	2.4	(0.7)
	**n**	**(%)**
Maternal country of origin		
Dutch	2,365	(50.2)
Non-Dutch	2,305	(49.8)
Maternal education level		
High	2,955	(56.6)
Middle	912	(30.6)
Low	803	(12.8)
	**Mean**	**(SD)**
Maternal age at pregnancy, y	29.9	(5.3)

Note: Numbers denote children included in 1 or more analyses.

Next, we tested the associations of gender diversity with experiences of loneliness in adolescence. Among AFAB youth, reports of gender-diverse experiences were concurrently associated with higher levels of loneliness (*B* = .67, 95% CI 0.49 to 1.23). Associations of a comparable size were observed between gender-diversity and loneliness experiences among AMAB adolescents (*B* = .45, 95% CI 0.32 to 0.98) (Table 2). Adjusting for anxiety and depression symptoms attenuated the associations (Table 3), but the associations remained significant in both AFAB (*B* = .38, 95%CI 0.24 to 0.51) and AMAB (*B* = .31, 95% CI 0.17 to 0.46) youth, respectively.

**Table 2:** Associations of Adolescents’ Report of Gender Diversity and Loneliness Experiences at Age 13 to 15 Years in Generation R Study (N = 4,424)

Adolescents with gender-diverse experiences (n = 187)	Self-reported loneliness		
	*B*	(95% CI)	*p*
Assigned sex at birth			
Assigned female			
Model 1	0.67	(0.49 to 1.23)	.001
Model 2	0.38	(0.24 to 0.51)	.001
Assigned male			
Model 1	0.45	(0.32 to 0.98)	.001
Model 2	0.31	(0.17 to 0.46)	.001

Note: Linear regression analysis of gender diversity with loneliness. B values are averaged from 20 imputed datasets. Model 1 is adjusted for child age and child puberty, maternal age, country of origin, and education. Model 2 is additionally adjusted for child anxiety and depression.

**Table 3:** Correlation Coefficients Between Loneliness With Anxiety and Depression

	1	2	3	4	5
1. Loneliness	—				
2. Depression	0.418^a^	—			
3. Anxiety	0.387^a^	0.603^a^	—		
4. Depression, mother report	0.246^a^	0.507^a^	0.340^a^	—	
5. Anxiety, mother report	0.196^a^	0.338^a^	0.427^a^	0.619^a^	—

Note: ^a^Correlation is significant at the *p* = .01 level (2-tailed).

As shown in Table 4, there was evidence for an interaction effect of gender diversity with being a victim of sibling bullying in relation to adolescent loneliness at age 15 (*p* = .04). The direction of this effect suggests that bullying by siblings (victimization) was more frequent in gender-diverse youth with higher levels of loneliness. The regression is decomposed into the main effects of gender diversity and bullying by siblings and interaction between them on adolescent loneliness. We observed direct associations of gender diversity and sibling victimization with higher levels of loneliness; however, the interaction between gender diversity and sibling victimization was not significant. Similarly, we observed an association between gender-diverse youth and maladaptive parenting (Table 5), but no interaction was found between gender diversity and maladaptive parenting on loneliness in adolescents.

**Table 4:** Interaction of Adolescents’ Report of Gender Diversity With Family Functioning Factors in Relation to Self-Reported Loneliness at Age 13 to 15 Years in Generation R Study (N = 4,424)

Adolescents with gender-diverse experiences (n = 187)	Self-reported loneliness		
	*B*	(95% CI)	*p*
**Model 1: Interaction effect between gender diversity and maladaptive parenting**			
Gender diversity	0.35	(0.23 to 0.51)	.001
Maladaptive parenting	0.23	(0.15 to 0.51)	.026
Gender diversity × maladaptive parenting	0.26	(−0.05 to 0.31)	.236
**Model 2: Interaction effect between gender diversity and being victimized by siblings**			
Gender diversity	0.93	(0.76 to 1.11)	.001
Victimization (bullied by a sibling at home)	0.13	(0.08 to 0.17)	.001
Gender diversity × victimization	0.21	(0.07 to 0.43)	.004
**Model 3: Interaction effect between gender diversity and being a perpetrator of sibling bullying**			
Gender diversity	0.92	(0.74 to 1.10)	.001
Perpetration (bullied a sibling at home)	0.26	(0.02 to 0.04)	.001
Gender diversity × perpetration	0.05		.133

Note: Interaction effect of gender diversity and family functioning factors on child loneliness. B values are averaged from 20 imputed datasets. Models are adjusted for child age and child puberty, maternal age, country of origin, and education.

**Table 5:** Associations of gender diversity and maladaptive parenting (N = 4,424)

Adolescents with gender-diverse experiences (n = 187)	Harsh parenting			Parenting overreactivity		
	*B*	(95% CI)	*p*	*B*	(95% CI)	*p*
Assigned sex at birth						
Assigned female	0.12	(0.07 to 0.29)	.004	0.13	(0.09 to 0.19)	.001
Assigned male	0.25	(0.14 to 0.47)	.019	0.21	(0.15 to 0.56)	.013

Note: Linear regression analysis of gender diversity, harsh parenting, and parenting overreactivity. *B* values are averaged from 20 imputed datasets. Models are adjusted for child age and child puberty, maternal age, country of origin, and education.

Further examination of child temperament showed an association between gender diversity and negative affect with less loneliness in adolescents (*B* = −2.67, 95% CI −4.81 to −0.18); however, we found no evidence for interaction effects between gender diversity and any temperamental traits on adolescent loneliness (Table 6).

**Table 6:** Interaction of Adolescents’ Report(−0.02 to 0.17) of Gender Diversity With Temperamental Traits in Relation to Self-Report Loneliness at Age 13 to 15 Years the Generation R Study (N = 4,424)

Adolescents with gender-diverse experiences (n = 187)	Self-reported loneliness		
	*B*	(95% CI)	*p*
**Model 1: Interaction effect between gender diversity and negative affect**			
Gender diversity	0.38	(0.22 to 0.49)	.001
Negative affect	−2.67		.032
Gender diversity × negative affect	−0.57	(−0.87 to 0.01)	.279
**Model 2: Interaction effect between gender diversity and surgency**			
Gender diversity	0.50	(0.35 to 0.82)	.010
Surgency	−3.12	(−4.36 to 0.09)	.651
Gender diversity × surgency	−0.21	(−4.36 to 0.09)	.445
**Model 3: Interaction effect between gender diversity and effortful control**			
Gender diversity	0.22	(0.13 to 0.46)	.009
Effortful control	0.18	(−0.02 to 0.35)	.113
Gender diversity × effortful control	0.10	(−0.06 to 0.04)	.534

Note: Interaction effect of gender diversity and temperamental traits on child loneliness. *B* values are averaged from 20 imputed datasets. Models are adjusted for child age and child puberty, maternal age, country of origin, and education.

A repeated-measures analysis examining changes in loneliness between ages 10 and 14 did not reveal a significant effect over time (*p* = .56). Additionally, we further explored the relation between pubertal development and subjective loneliness. These analyses suggest that adolescents who are undergoing pubertal changes report higher levels of loneliness (*B* = .18, 95% CI 0.15 to 0.24).

Sensitivity analyses were performed to assess potential bias due to sample restriction. First, we compared demographic characteristics and key study variables between the full sample (N = 4,424) and the gender-diverse analytic subsample (n = 187); distributions were broadly similar, suggesting minimal selection bias. Second, results were consistent when analyses were repeated including adolescents with partially missing pubertal data. These findings indicate that restricting the sample did not alter the observed associations.

## Discussion

We used a large population-based study to examine the associations of gender diversity with loneliness experiences in adolescence. In addition, we examined the interaction effects between family environmental factors, temperamental traits, and gender diversity in relation to adolescent loneliness. We highlight 3 key findings. First, our results suggest that adolescents who reported gender diversity had higher levels of loneliness compared with youth who did not report gender diversity. Associations between gender diversity and loneliness in adolescents did not differ by child sex assigned at birth. The associations remained when we accounted for depression and anxiety, indicating a unique association of gender diversity with lonely feelings in adolescence. Second, being bullied by a sibling at home (victimization) modified the association of gender diversity with loneliness experiences, suggesting that gender-diverse youth with higher levels of loneliness are likely to be bullied by a sibling at home. Third, we found no evidence for interaction effects between gender diversity, maladaptive parenting, and any temperamental traits on adolescent loneliness.

Loneliness can affect individuals at any age as they experience challenges negotiating each life stage. Loneliness may have its onset earlier or later in life, and it may be transient or persistent in duration. Adolescence is a key stage for identity development (Erikson’s identity vs role confusion), which paves the way to form intimate relationships (Erikson’s intimacy vs isolation).^63^ For gender-diverse youth, loneliness can disrupt these processes by hindering self-identity and social connections. The minority stress model highlights how discrimination and rejection amplify loneliness in this group, making it a significant barrier to emotional and social development.^12^ Negotiating this period is even more challenging for gender-diverse youth, who have been shown to experience higher levels of loneliness.^64^^,^^65^ Our findings also support increased levels of lonely feelings in gender-diverse adolescents. Accordingly, loneliness could be especially problematic for gender-diverse individuals in this developmental period. Although inquiries into loneliness, as an experience separate from depression and anxiety, should be evaluated in all youth, clinicians should be aware that gender-diverse youth have higher rates of loneliness.

Given our cross-sectional design, we tested depression and anxiety as potential confounders rather than mediators, as testing mediation would require longitudinal data to establish temporal ordering. It is also important to acknowledge that most of the youth in our study are not being seen in a clinical setting except for routine care. Thus, primary care physicians should be aware of the extra challenges that these youth face and be willing to provide a safe place to explore gender diversity, bullying, and mental health symptoms during routine visits. For youth who are struggling, cognitive-behavioral therapy has shown promise in addressing loneliness and mental health concerns,^66^ whereas peer support programs and family acceptance initiatives, such as the Family Acceptance Project, may help reduce isolation.^32^^,^^67^ School-based programs promoting inclusivity and anti-bullying policies can also create safer environments.^68^ Although these interventions are supported by research, further studies are needed to determine their effectiveness specifically for gender-diverse youth, taking into account the full spectrum of intersecting identities and community contexts.

The vulnerability of gender-diverse individuals to sibling bullying is consistent with previous studies reporting that gender-diverse individuals are substantially more likely to experience sibling bullying^69^^,^^70^ compared with those who did not report gender diversity. For example, cisgender female and nonbinary youth who were AFAB were more likely to report sibling bullying compared with cisgender male youth. Moreover, youth who were AMAB but presented traditionally feminine characteristics were also at risk of being bullied by siblings.^69^ Importantly, we found that gender diversity interacts with sibling bullying (victimization) in association with loneliness in adolescence. Although it appears that gender diversity plays a role in sibling bullying, our findings regarding the associations between gender diversity and sibling bullying (victimization) with loneliness did not differ by sex assigned at birth. Next, we found a main effect for the association between gender diversity and bullying a sibling at home (perpetration), but no interaction effects in association with loneliness, suggesting that gender-diverse youth experience relatively high levels of loneliness, regardless of whether they are likely to bully a sibling at home (perpetration).

Sibling bullying, often overlooked in research, may significantly contribute to loneliness and mental health challenges among gender-diverse youth. Our findings suggest that addressing family dynamics, including sibling interactions, could improve clinical interventions. For example, family therapy focused on communication and conflict resolution may help reduce the negative impact of sibling bullying. Although peer relationships become more central during adolescence, the family environment remains critical as youth develop autonomy. More research is needed to explore the full scope of family support, particularly sibling bullying, to inform tailored interventions.

Gender-diverse youth, whether exploring or settled in their gender identity, face different challenges. The exploratory phase, marked by uncertainty and social stigma, requires additional support. Recognizing this diversity is key to developing inclusive strategies. Preventive measures, such as support groups for siblings of transgender and nonbinary youth, can foster understanding, reduce bullying, and improve family support, helping alleviate loneliness for both adolescents and their siblings.

Contrary to our hypothesis, we were unable to demonstrate an interaction effect between gender diversity and maladaptive parenting or temperamental traits in the association with adolescents who experienced loneliness. However, we found that gender diversity and negative affectivity were each associated with higher levels of loneliness in adolescence. Although gender diversity and maladaptive parenting were each associated with higher levels of loneliness in adolescents, we found no evidence of an interaction. Research suggests that gender diversity affects all members of the family in unique, highly personal, and various ways, which are not always readily apparent to others. Evidence of the lack of parental support on the mental health, resilience, and quality of life of gender-diverse youth has been well described in the literature.^71^^,^^72^

Our temperament measures may not fully capture how gender diversity interacts with loneliness, possibly because other factors, such as peer support or identity affirmation, are also at play. Much of the existing research on temperament and loneliness has focused on gender-typical youth, so future studies should examine these dynamics in more diverse populations. Research from the Family Acceptance Project and the Trevor Project underscores the importance of professional support and parental acceptance in fostering positive outcomes for gender-diverse youth.^73^^,^^74^ Katz-Wise *et al.*^72^ recommended that health care professionals address family acceptance and rejection during routine clinical visits. Since then, several efforts have been made to integrate these discussions into clinical practice. For example, the Family Acceptance Project, a long-standing research-to-practice initiative, has expanded its evidence-based family support model across clinical settings in the United States and internationally, offering training, resources, and community outreach to help providers and families foster acceptance and reduce mental health risks for gender-diverse youth.^32^ Clinical guidelines increasingly emphasize affirming family engagement as part of routine care for gender-diverse youth.^75^^,^^76^ These efforts have shown promise in improving family dynamics and supporting mental health. The absence of an interaction effect may reflect the independent influence of gender diversity, maladaptive parenting, and temperamental traits on loneliness, or an effect may emerge only under specific conditions not captured in the current study. Future research could explore the conditions and moderating factors under which these variables interact.

Moreover, when further exploring the relation between pubertal development and subjective loneliness, we found that pubertal development was associated with higher levels of loneliness in adolescence. These findings highlight the potential emotional challenges faced by adolescents during pubertal development. The increased loneliness observed may suggest a need for targeted interventions or support systems to address the psychological well-being of adolescents in this developmental stage.

The current study has several limitations. First, this study was cross-sectional and lacked repeated measures of gender diversity and loneliness. Second, our measure of gender diversity did not adequately cover the full spectrum, as we relied on only 2 items and did not directly measure gender identity, gender expression, or gender dysphoria. In addition, we did not account for participants who had socially or medically transitioned. Moreover, loneliness was measured using a series of primary and secondary questions that were then coded into a single item. Insofar as the measure is limited, it may underestimate the effect of gender diversity on loneliness outcomes. By incorporating more inclusive gender identity options, researchers can better understand and address the specific health challenges faced by nonbinary and transgender individuals, ultimately leading to more tailored and effective interventions. A further limitation is that the gender-related items on the YSR and CBCL do not differentiate between gender role and gender identity. Consequently, our measure may capture variation in gender expression that does not necessarily reflect differences in gender identity, which could affect the interpretation of associations with loneliness.

Another limitation of this study is the use of maternal reports, which may not fully capture caregiver variability. Future research should consider using multiple informants and differentiating between data from primary and maternal caregivers to explore potential effects on gender identity development. Moreover, a limitation of the current study is the reliance on proxy measures from parents for racial/ethnic identity of youth. This indirect observation, though common in large cohort studies, may not fully capture adolescents’ self-perceived identities. Finally, adolescents were not asked about how they perceived and experienced their parent’s parenting style. Following the methodology of the Generation R Study, data in the current study were primarily obtained from the gestational/female-identifying parents, a convenience-driven approach that may limit the diversity of caregiver perspectives. However, previous research suggests that including data from both parents or all caregivers is unlikely to significantly alter the primary outcomes.^77^

Study strengths include a large population-based sample in which the mothers were recruited during pregnancy or at birth and a broad spectrum of measured covariates. Further, we included both maternal and paternal reports on child gender diversity and therefore could investigate combined parent and offspring reports on gender diversity and loneliness experiences.

Although external stressors, such as discrimination and internalized transphobia, are important contributors to mental health challenges faced by gender-diverse individuals, loneliness is a crucial factor that requires specific attention, especially in gender-diverse youth.^78^ Loneliness can arise as an emotional response to external stressors, exacerbating feelings of isolation, and can contribute to cycles of depression, anxiety, and withdrawal. Addressing loneliness is essential, not only because it impacts mental well-being, but also because it impedes coping strategies and resilience.^79^ Moreover, reducing loneliness, especially during adolescence, and fostering social connection can act as a buffer against the negative effects of external maltreatment, providing a pathway to greater emotional well-being.^80^ In this way, targeting loneliness offers a unique intervention point that addresses both the emotional and the psychological needs of gender-diverse individuals, ultimately improving their overall mental health.

In summary, we found that gender diversity is associated with higher levels of loneliness in adolescents. Moreover, being a target of bullying modified the association of gender diversity with loneliness experiences, suggesting that gender-diverse adolescents who are being bullied by siblings experience particularly higher levels of loneliness. The cross-sectional nature of the study does not allow us to infer causality, and thus it may be that greater feelings of loneliness in gender-diverse youth may result in being bullied by siblings, for instance, because the loneliness results in withdrawn behavior and lack of bonding. No such interaction was found for gender diversity and bullying siblings, maladaptive parenting, and any of the temperamental traits with loneliness experiences in adolescence. Clinicians who are seeing gender-diverse youth should inquire about symptoms of loneliness and being the victim of bullying by siblings. Future studies should examine whether sibling support, independent of bullying, contributes to resilience in gender-diverse youth, potentially offering unique benefits beyond peer relationships.

## CRediT authorship contribution statement

**Yllza Xerxa:** Writing – original draft, Formal analysis, Conceptualization. **Akhgar Ghassabian:** Supervision, Resources, Conceptualization. **Manon H.J. Hillegers:** Writing – review & editing. **Luis Martinez Agulleiro:** Writing – review & editing. **Pauline W. Jansen:** Writing – review & editing. **Samantha Busa:** Writing – review & editing. **Francisco Xavier Castellanos:** Writing – review & editing. **Tonya White:** Writing – review & editing, Supervision, Conceptualization.
