# Supplementary material for: Loneliness and Bullying by Siblings in Gender-Diverse Adolescents: Results From the Population-Based Generation R Study
Source: JAACAP Open. 2026 Apr 23;4(4):589–600. doi: 10.1016/j.jaacop.2026.04.003 (PMC13420606; doi:10.1016/j.jaacop.2026.04.003)
Supplement: Supplemental Material [file mmc1.docx]

***Supplementary material***

**METHODS**

***Gender diversity***

We selected items from the Child Behavior Checklist forms and the Gender Identity/Gender Dysphoria Questionnaire for Adolescents and Adults (GIDYQ)^1^ to assess gender diversity in adolescents. From the Child Behavior Checklist (CBCL/6-18, reported by parent) and the Youth Self-Report (YSR/11-18, reported by child) of ASEBA,^2,3^ we used item #110: ‘Wish to be the opposite sex’, with choices 0 = ‘not true’, 1 = ‘somewhat or sometimes true’, and 2 = ‘very true or often true’, based on the preceding two months. From the GIDYQ, we selected the question: ‘Would you rather be treated as someone from the opposite sex’ Adolescents responded ‘no’, ‘probably yes’, or ‘definitely yes’. While we used both self and parent reports of gender diversity, the number selected by parent report was low (only 31 participants (16.6%) were identified as gender-diverse via parent-report, compared to 156 (83.4%) via self-report).^4^ We dichotomized the responses by combining ‘somewhat or sometimes true’ and ‘very true or often true’ and ‘probably yes’ and ‘definitely yes’ for the ASEBA and GIDYQ, respectively, and defined gender-diverse adolescents if any item was endorsed by parents and/or adolescents.^4^ In our study, adolescents were not specifically asked whether they identified as transgender or if they had socially transitioned. Additionally, data were collected through self-report at this age, which allowed us to capture the adolescents’ own experiences and self-perceptions during a critical time of identity exploration, even though this covers a period of adolescent exploration and their gender identity may not yet be fully solidified. While we recognize that gender identity may be fixed at younger ages for some individuals, whereas for others, gender identity exploration can continue into adulthood, we focused on the adolescent developmental window due to its relevance in the broader social context of embodiment and gendered experiences that are visible to youth and peers alike. We acknowledge the variability in neurodevelopmental trajectories, particularly among transgender youth, and that these factors may influence the timing and experience of gender identity exploration. Therefore, the age range in this study represents one epoch within the broader spectrum of gender identity development.

While gender exploration can be an important stage of gender identity development, it is distinct from a persistent and enduring identification with a gender different from the one assigned at birth. The youth included in this study may be in various stages of gender exploration, which can present different mental health and social challenges than those faced by youth who have a persistent, long-term identification with a gender different from their assigned sex. This distinction is important when considering the unique needs of gender-diverse youth and further research is needed to understand how these different experiences may influence mental health and well-being.

Gender identity is a complex, multifaceted experience that may unfold over time. While some youth identify as a gender different from their sex assigned at birth for as long as they remember, for others, their gender diversity or gender questioning can emerge during adolescence.^5^ The process of exploring gender identity is fluid and varies across individuals. This paper recognizes that not all gender-diverse youth will have a persistent, stable gender identity.

**Adolescent loneliness**

Loneliness was assessed by a collection of measures using the self-reported Brief Problem Monitor (BPM) at age 10 and the Youth Self-Report (YSR/11-18)^2,3^ at age 14. Each item is scored on a three-point rating scale with: 0 = ‘not true', 1 = 'somewhat or sometimes true', and 2 = ‘very true or often true', based on the preceding two months. Youth reported on 4 items that measure loneliness: ‘*I like being with people’ (reverse coded)*, ‘*I feel lonely’*, ‘*I would rather be alone than with others’,* ‘*Other boys or girls don't like me’.* The internal consistencies (Cronbach’s alpha) for loneliness score was 0.61. The continuous loneliness scale was used in the analyses, which is the mean score of all items.

These items index both a perceived quantitative lack of contacts in one's social network and a perceived qualitative deficit in existing relations. As such, this construct is best considered as perceived social isolation (feeling lonely) as opposed to being alone.

**Depression and anxiety**

Information on offspring depression and anxiety was obtained using the Brief Problem Monitor (BPM/11-18),^6,7^ a standardized child self-report of problem behaviors (ages 10 and 15). The BPM/11-18 is a validated abbreviated version of the Youth Self-Report (YSR/11-18).^2,3^ Each item is scored on a three-point rating scale with: 0 = 'not true', 1 = 'somewhat or sometimes true', and 2 = 'very true or often true', based on the preceding two months. The depression and anxiety subscale comprised items such as: “I am unhappy, sad or depressed” and “I am too anxious or fearful”. For each subscale, we computed a continuous total score, which is the mean score of all items within the subscale.

**Maladaptive parenting (parent report)**

Maladaptive parenting was assessed by a collection of measures using the Overreactivity subscale from the Parenting Scale and using an adapted version of the Parent-Child Conflict Tactics Scale (CTSPC).^8^ The Overreactivity subscale was administered to measure emotional reactivity in the context of discipline encounters (e.g., ‘Things build up and I do things I don’t mean to’, ‘I usually get into a long argument with my child, and’ I give my child a long lecture’). This subscale consists of 6 items reflecting mistakes, such as displays of anger, meanness, and irritability, which are rated on a six-point rating scale (in the past year; 1 = 'never', 2 = 'once', 3 = 'two times', 4 = ‘three times’, 5 = ‘four times’, 6 = ‘more than 4 times’). The Overreactivity subscale exhibited adequate internal consistency and test-retest reliability (.82).^9^ Harsh parenting contained 10 items on different discipline tactics during the past 2 weeks on a six-point rating scale (1 = ‘never’, 2 = ‘once’, 3 = ‘two times’, 4 = ‘three times’, 5 = ‘four times’, 6 = ‘more than four times’). Discipline tactics varied from explaining why something was wrong, distracting, punishing to, and verbal or physical aggression. Example items are ‘I punished him/her by forbidding something that he/she wanted to do or have’ and ‘I pinched his/her arm angrily’.

Three items of the minor Physical Assault Scale of the CTSPC on hitting and spanking (e.g., ‘hit child on the bottom with something like a belt, stick or some other hard object’) were not assessed. These items were omitted because all forms of corporal punishment against children including spanking, hitting or using implements have been explicitly prohibited under Dutch law since 2007, as part of amendments to the Civil Code that affirm children’s inviolability and physical integrity. A harsh parenting subscale can be calculated by summing the following 6 items: ‘In the past week/month, I angrily pinched my child’s arm’, ‘I shouted, yelled or screamed angrily at my child’, ‘I scolded at my child’, ‘I threatened to slap, spank or hit my child but did not actually do it’, ‘I called my child dumb or lazy or some other name like that’ and ‘I shook my child’, with higher scores reflecting harsher discipline style.^10^ In the current sample, internal consistency (Cronbach’s alpha) was 0.63 for harsh parenting and 0.75 for over-reactivity. These values are comparable to those reported in the scale development study (α = 0.69 and α = 0.74, respectively).^11^

**Bullying and victimization by siblings**

When children were 13 - 15 years old, they were asked to report their involvement in sibling bullying. Items were based on a sibling bullying questionnaire adapted from the Olweus Bullying Questionnaire,^12^ addressing bullying between brothers and sisters.^13^ Children were told that sibling bullying is: when a brother or sister tries to upset you by saying nasty and hurtful things, or completely ignores you from their group of friends, hits, kicks, pushes or shoves you around, tells lies or makes up false rumors about you. They were then asked to report on their experience of sibling bullying within the last 6 months. Children were first asked to report on 4 items, assessing whether they were ever bullied by a sibling at home (victimization by a sibling), for example, “How often in recent months has your sibling beaten, kicked or pushed you?”. Four parallel items asked whether they had ever bullied a sibling at home (perpetrator of sibling bullying) on a 5-point Likert scale (0 = never ; 1 = only ever once or twice ; 2 = 2 or 3 times a month ; 3 = about once a week ; 4 = several times a week), for example, “How often in recent months have you beaten, kicked or pushed your sibling?”. For this study, we computed two continuous sum scores, representing the mean score of all items within each subscale (victimization and perpetration). The internal consistency (Cronbach’s alpha) for sibling victimization score was 0.78, and for perpetration sibling score was 0.76.

**Temperament**

Child temperament was assessed by the Children’s Behavior Questionnaire -Very Short Form (CBQ-VSF)^14^ when children were 6 years old. For consistency and reliability, only data from female-identifying parents (predominantly mothers) were included, as they were the primary caregivers in the vast majority of participating families and were the most consistent informants across study waves. This approach aligns with previous research using parent-report measures in similar populations. The CBQ-VSF provides a highly differentiated assessment of temperament that has been validated across various studies.^14^ The CBQ-VSF consists of 36 items informing about child behavior in daily situations. Mothers rated children’s reactions in the past 6 months on a 7-point Likert scale (1 = extremely untrue to 7= extremely true). Temperament refers to biologically based individual differences in emotional, motor, and attentional reactivity and self-regulation, which are relatively stable over time and observable early in life. The CBQ-VSF includes three subscales: (1) negative affect consists of 12 items, e.g., “gets quite frustrated when prevented from doing something s/he wants to do”, negative affect refers to negative emotional reactivity such as anger, frustration, sadness, and fear; (2) surgency consists of -12 items, e.g., “is full of energy, even in the evening”, surgency is measured with items of behavioral activation such as impulsivity, activity level, high-intensity pleasure, and motor activation.; and (3) effortful control consists of 12 items, e.g., “when building or putting something together, becomes very involved in what s/he is doing, and works for long periods”, effortful control is assessed with questions on regulatory behaviors such as inhibitory control and attentional focusing and shifting.^14^ The scores of negative affect, surgency, and effortful control were computed by averaging the item scores. The internal consistencies (Cronbach’s alpha) for negative affect was 0.74, for surgency 0.74 and for effortful control 0.71.

***Covariates***

Date of birth (used to calculate a child’s age) and assigned sex assigned at birth were obtained from birth records. Maternal and paternal age were assessed at enrollment during pregnancy. In terms of effects related to ethnographic identity, the current study centered migrant generation. Maternal country of origin was categorized as Dutch and non-Dutch. Information on country of origin was obtained from female-identifying parents only, as this data was collected at enrollment during pregnancy and thus primarily reflects maternal background. To facilitate comparison with previously collected data, we used maternal national origin as a proxy for child ethnicity. This variable was based on the mother’s country of birth and her parents’ country of birth, as race or ethnicity were not directly assessed in the way commonly defined in U.S. based research. Given the diverse population of Rotterdam and its surroundings, participants reported a wide range of ethnic backgrounds, including Dutch, Moroccan, Turkish, Surinamese, Cape Verdean, Dutch Antillean, and various other non-Dutch European, Asian, African, and American origins. Due to small sample sizes in several subgroups, maternal national origin was operationalized as a two-category variable: Dutch and Non-Dutch (including European (non-Turkish), Turkish, Moroccan, Surinamese, and Other Ethnicity/National Origin). Maternal education was classified in three levels: ‘low’ (maximum of three years general secondary school), ‘medium’ (>3 years general secondary school; intermediate vocational training), and ‘high’ (Bachelor’s degree or higher academic education). Maternal education may reflect socioeconomic status, health literacy, and access to resources, all of which can independently influence the exposure and outcome variables in our study. Similarly, maternal national origin, used here as a proxy for child ethnicity, captures broader social, cultural, and structural factors that may affect parenting practices, access to care, and experiences of discrimination or systemic inequality. Failing to adjust for these variables could obscure the associations of interest by attributing variance explained by these background factors to the primary exposures under investigation. In questionnaires collected at the 13-15-year-old wave, puberty was assessed via self-report using the Puberty Developmental Scale,^15^ a validated self-report questionnaire with 5 items answered on a five-point scale.

**References:**

1. Deogracias JJ, Johnson LL, Meyer-Bahlburg HF, Kessler SJ, Schober JM, Zucker KJJJosr. The gender identity/gender dysphoria questionnaire for adolescents and adults. 2007;44(4):370-379.

2. Achenbach, Rescorla. *Manual for the ASEBA preschool forms & profiles: An integrated system of multi-informant assessment; Child behavior checklist for ages 1 1/2-5; Language development survey; Caregiver-teacher report form*. University of Vermont; 2000.

3. Achenbach, Rescorla. ASEBA school-age forms & profiles. Aseba Burlington, VT; 2001.

4. Ghassabian A, Suleri A, Blok E, Franch B, Hillegers MHJ, White T. Adolescent gender diversity: sociodemographic correlates and mental health outcomes in the general population. *Journal of child psychology and psychiatry*. 2022;

5. Cohen-Kettenis PT, Pfäfflin F. *Transgenderism and intersexuality in childhood and adolescence: Making choices*. vol 46. Sage; 2003.

6. Achenbach TM, McConaughy SH, Ivanova MY, Rescorla LA. Manual for the ASEBA brief problem monitor (BPM). *Burlington, VT: ASEBA*. 2011:1-33.

7. Achenback TM, Rescorla LA. Manual for the ASEBA school-age forms & profiles. *Burling: University of Vermont (Research center for children, youth and families)*. 2001;

8. Straus MA, Hamby SL, Finkelhor D, Moore DW, Runyan D. Conflict Tactics Scale: Parent to Child. *Assessment of family violence: A handbook for researchers and practitioners*. 1998;

9. Arnold DS, O'Leary SG, Wolff LS, Acker MM. The Parenting Scale: a measure of dysfunctional parenting in discipline situations. *Psychological assessment*. 1993;5(2):137.

10. Jansen PW, Raat H, Mackenbach JP, et al. Early determinants of maternal and paternal harsh discipline: The generation R study. *Family relations*. 2012;61(2):253-270.

11. Arnold DS, O'Leary SG, Wolff LS, Acker MM. The Parenting Scale: A measure of dysfunctional parenting in discipline situations. doi:10.1037/1040-3590.5.2.137. *Psychological Assessment*. 1993;5(2):137-144. doi:10.1037/1040-3590.5.2.137

12. Olweus D. Olweus bullying Questionnaire Center City. *MN: Hazelden Foundation*. 2007;

13. Dantchev S, Wolke D, Zammit S. Sibling bullying in middle childhood and psychotic disorder at 18 years: a prospective cohort study. *Psychological Medicine*. 2018;48(14):2321-2328. doi:Doi: 10.1017/s0033291717003841

14. Putnam SP, Rothbart MK. Development of short and very short forms of the Children's Behavior Questionnaire. *J Pers Assess*. Aug 2006;87(1):102-12. doi:10.1207/s15327752jpa8701_09

15. Carskadon MA, Acebo C. A self-administered rating scale for pubertal development. *Journal of Adolescent Health*. 1993/05/01/ 1993;14(3):190-195. doi:<https://doi.org/10.1016/1054-139X(93)90004-9>
